# Mesenchymal stem cell-derived extracellular vesicles for treatment of bone loss within periodontitis in pre-clinical animal models: a meta-analysis

**DOI:** 10.1186/s12903-023-03398-w

**Published:** 2023-09-29

**Authors:** Huan Zhou, Yan-Xin Qi, Chun-Hui Zhu, Ang Li, Dan-Dan Pei

**Affiliations:** 1 Key Laboratory of Shaanxi Province for Craniofacial Precision Medicine Research, College of Stomatology, Xi’an Jiaotong University, Xi’an, China; 2 Department of Periodontology, College of Stomatology, Xi’an Jiaotong University, Xi’an, China; 3 Department of Prosthodontics, College of Stomatology, Xi’an Jiaotong University, Xi’an, China

**Keywords:** Mesenchymal stem cell, Extracellular vesicle, Periodontitis, Bone regeneration

## Abstract

**Background:**

Mesenchymal stem cell-derived extracellular vesicles (MSC-EVs) represent an effective and promising strategy for periodontitis, although studies remain pre-clinical. Herein, a meta-analysis was conducted to assess the efficacy of MSC-EVs in animal models of periodontitis.

**Methods:**

The PubMed, Web of Science, and Embase electronic databases were searched up to Dec 2022 to retrieve preclinical studies examining the use of MSC-EVs for periodontitis treatment. Meta-analyses and sub-group analyses were performed to assess the effect of MSC-EVs on Bone Volume/Total Volume (BV/TV) or the distance between the cementoenamel junction and alveolar bone crest (CEJ-ABC) in pre-clinical animal models of periodontitis.

**Results:**

11 studies published from Mar 2019 to Oct 2022 met the inclusion criteria. Overall, MSC-EVs contributed to periodontal bone regeneration in the inflammatory bone loss area due to periodontitis, as represented by a weighted mean difference (WMD) of 14.07% (95% CI = 6.73, 21.41%, *p* < 0.001) for BV/TV and a WMD of -0.12 mm (95% CI= -0.14, -0.11 mm, *p* < 0.001) for CEJ-ABC. However, sub-analysis suggested that there was no significant difference in CEJ-ABC between studies with bioactive scaffolds and studies without bioactive scaffolds (*p* = 0.60).

**Conclusions:**

The present study suggests that MSC-EVs may represent an attractive therapy for the treatment of inflammatory bone loss within periodontitis.

**Supplementary Information:**

The online version contains supplementary material available at 10.1186/s12903-023-03398-w.

## Introduction

Periodontitis is a widespread disease that affects almost half of the adult population worldwide, and is characterized by connective tissue attachment loss and alveolar bone destruction, with subsequent tooth loss [1, 2]. In addition, toxins and pathogenic factors produced by periodontal pathogens are vital risk factors for a variety of systemic diseases, such as Alzheimer’s disease, diabetes, cardiovascular disease, cancer and autoimmune diseases, which seriously affect the quality of human life [3–5]. Therefore, the prevention and treatment of periodontitis is an intractable problem that needs to be solved. Over the past few decades, the strategy of stem cell-based periodontal regeneration has shed new light on facilitating new tissue in periodontal pockets [6, 7]. However, the clinical translation of mesenchymal stem cells (MSCs), such as periodontal ligament stem cells (PDLSCs), in patients suffering from periodontitis has been disappointing up to now [8, 9]. Analyses of the causes of stem cell transplantation failure in the clinic have shown that stem cell transplantation involves complicated operation measures and clinical transformation issues, including clinical preparation, cell storage and transportation, survival and viability of stem cells, and potential immune rejection after allogeneic cell implantation [10, 11]. Moreover, because the intrabony periodontal defect is an inflammatory microenvironment, the transplanted stem cells may be functionally compromised, along with other not fully clarified reasons and risks, such as aberrant differentiation and reduced stemness, thus dampening the enthusiasm of researchers to try to use stem cell transplantation directly [9, 12, 13].

Based on the current understanding, transplanted stem cells contribute to tissue regeneration and repair mainly via their paracrine functions. These paracrine secretions include a variety of growth factors, chemokines, cytokines and extracellular vesicles (EVs). These agents have been well demonstrated to regulate tissue regeneration by mobilizing or regulating the bioactivity of resident cells and modulating the microenvironment of the implantation area. EVs, particularly, have been regarded as the principal element contributing to the therapeutic efficacy of parent cells [14]. EVs are cell-secreted nanovesicles with the size ranging from 30 to more than 1000 nm, and among them, typical small EVs with the size of 30–150 nm are referred to as “exosomes” [15]. In fact, EVs contribute to intercellular communication by delivering specific agents to target cells or tissues for their loaded cargos, including various bioactive molecules in terms of mRNAs, lipids, proteins, DNA, circRNAs and microRNAs [16]. Current studies have confirmed that EVs participate in various biological and pathological processes, such as antigen presentation, coagulation process, virus transmission, tumor growth, and tissue regeneration [17]. More importantly, growing evidence has demonstrated that EVs may represent a promising therapeutic approach due to their advantages over donor cells, in terms of low propensity to initiate immune rejection after allogeneic administration, no risk of aneuploidy and high stability. In addition, it was reported that EVs can be stored for a long time and remain stable because their special membranous structure can prevent EVs from being degraded by enzymes [18–20]. Under this context, increasing numbers of studies have demonstrated that EVs secreted by stem cells may represent a promising and attractive approach for the treatment of periodontitis [13, 21].

Although accumulating evidence indicates that MSC-EVs may be a viable ready-to-use and cell-free therapeutic strategy for the treatment of periodontal defects [8, 12, 13], relevant clinical studies are lacking to date, and pre-clinical animal models remain the main source of study evidence, which has contributed prominently to the current understanding of periodontitis. Additionally, these pre-clinical models have been the spearhead to explore new therapeutic strategies for periodontitis. Thus, the principal objective of the present meta-analysis was to assess the efficacy of MSC-EVs in treating inflammatory bone loss in preclinical periodontitis models, with the aim of providing the most recent available evidence for MSC-EVs in periodontitis and promoting the translation of this novel cell-free therapy towards clinical trials for the treatment of periodontitis.

## Methods

### Protocol and registration

The present study was registered in the International Prospective Register of Systematic Reviews (PROSPERO, https://www.crd.york.ac.uk/prospero/, CRD42023385352), and the systematic review and meta-analysis were performed in accordance with the Preferred Reporting Items for Systematic Reviews and Meta-analyses (PRISMA) guidelines.

### Search strategy

Online databases in terms of PubMed, EMBASE and the Web of Science were searched for research articles published in English that examined the use of MSC-EVs to treat periodontitis. The search terms including the following: (“extracellular vesicle” or “exosome” or “EV” or “micro vesicle” or “microparticle” or “micro-vesicle” or “small extracellular vesicle” or “microvesicle” or “MV”) and (“stem cell” or “mesenchymal stem Cell” or “MSC”) and (“periodontitis” or “periodontal disease” or “periodontal defect” or “periodontal regeneration” or “periodontal bone loss” or “alveolar bone defect”). The search strategies were modified according to database and used filters for preclinical animal models. In addition, the reference lists of the included studies were manually searched to identify potentially relevant studies. For the present study, we followed the PICOS structure, population (P): animal models of periodontitis, with no restrictions on species or modeling approaches; Intervention (I): EVs derived from MSCs; Comparison (C): pre-clinical models of periodontitis treated without MSC-EVs; Outcome (O): Bone Volume/Total Volume (BV/TV), the distance between the cementoenamel junction and alveolar bone crest (CEJ-ABC) and signaling pathways involved; Study design (S): randomized, non-randomized or quasi-randomized in vivo studies.

### Eligibility criteria

The inclusion criteria were as follows: (1) studies on pre-clinical models of periodontitis; (2) studies on EVs derived from MSCs; (3) randomized, non-randomized or quasi-randomized in vivo studies; (4) studies comparing the effects of MSC-EVs with controls in pre-clinical models of periodontitis; and (5) studies published in English and full-text available.

The exclusion criteria were as follows: (1) studies on EVs derived from other cells instead of MSCs or other tissues in periodontitis; (2) studies without in vivo models and results; (3) studies exploring the function of MSCs or their conditioned medium in periodontitis; (4) studies with insufficient information or data that could not be fully extracted; and (5) non-comparative studies, case reports, review articles, commentaries, conference proceedings, letters to the editor, abstract editorials, monographs, and other study types.

### Literature search and study selection

A systematic literature search of all potential studies was performed to identify all the relevant studies (published up to Dec, 2022). In specific, two reviewers (HZ, YXQ) independently searched the databases and then excluded the duplicates. Then, three investigators (YXQ, DDP, AL) screened and evaluated the research titles and abstracts based on the eligibility criteria. The remaining studies that appeared to meet the aforementioned inclusion criteria were then subjected to full-text screening. Any disagreements regarding inclusion were resolved through discussion with all reviewers until a consensus was achieved.

### Data extraction

Two authors (HZ, YXQ) extracted the relevant data from the included studies independently using a specific form (Excel, Microsoft, Seattle, USA), and then, all the authors re-checked and confirmed the obtained raw data. The following data were extracted from each study: (1) MSC source, MSC-EV isolation method and characterization, and the administration dose of MSC-EVs; (2) study design, i.e., the comparator information, animal species, sample size, and administration route; (3) intervention characteristics, i.e., delivery strategies in terms of hydrogels and/or scaffolds and others, modification to MSC-EVs; (4) outcomes i.e., BV/TV, CEJ-ABC, and signaling pathways involved; and (5) study characteristics, i.e., authors, location of the research, and publication year. Data were acquired from the presented graphs with the help of OriginPro2021(Version: 9.8.0.200) when the raw data were not provided.

### Outcome measures

To assess the function of MSC-EVs in the treatment of bone loss within periodontitis, the primary outcome measure was to compare the BV/TV, CEJ-ABC changes in treating inflammatory bone loss in periodontitis models with the control groups. The secondary outcome measure was the signaling pathways involved in the MSC-EVs for treatment of bone loss within periodontitis.

### Quality assessment

Risk of bias assessment for studies was carried out according to the guidelines of the Systematic Review Centre for Laboratory Animal Experimentation (SYRCLE) bias risk tool [22]; specifically, for each study, the selection bias, performance bias, detection bias, attrition bias, reporting bias and other sources of bias were assessed by two reviewers (YXQ, CHZ) independently. For each domain, the risk of bias was categorized as high, low or unclear (if the risk of bias could not be assessed due to a lack of sufficient information). Any disagreement regarding the risk of bias was resolved through discussion with a third reviewer (DDP).

### Data analysis

For the present meta-analysis, the combined effect size was expressed as the weight mean difference (WMD) with a 95% confidence interval (CI). *p* < 0.05 (two-tailed) was considered to be statistically significant. The *I*^2^ statistic was performed to quantify the heterogeneity across studies; specifically, *I*^2^ values indicating low, moderate and high heterogeneity with the thresholds of 25%, 50% and 75%, respectively [23]. In addition, sensitivity and sub-analyses were conducted to explore the sources of heterogeneity and to identify other potentially confounding factors. Publication bias was assessed with a funnel plot; besides, as a quantitative complement for the funnel plot, Egger’s test was also performed to evaluate the *P* value. Stata 15.1 (StataCorp, College Station, TX, USA) and RevMan 5.4.1 software (Cochrane Collaboration; www.cochrane.org/) were utilized for data analyses.

## Results

### Literature search & study characteristics

**Fig. 1:**
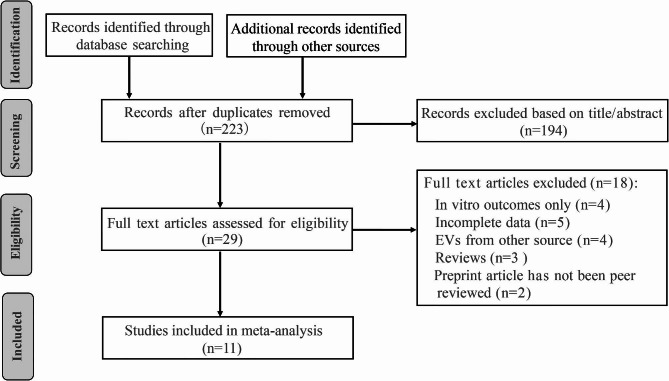
Flowchart of the enrolled studies on mesenchymal stem cell-derived extracellular vesicle (MSC-EV) therapy in periodontitis animal models

The study selection process is illustrated in Fig. 1. Specifically, 223 articles were initially identified based on the search strategy. After the initial review based on title/ abstract reading (Supplement file Table S1), 29 articles remained for further assessment. Then, 18 articles were excluded based on the inclusion and exclusion criteria, specifically, in vitro results only (*n* = 4), incomplete data (*n* = 5), EVs secreted from other cells instead of MSCs (*n* = 4), review articles (*n* = 3), and preprint articles that have not been peer reviewed (*n* = 2). Ultimately, the present meta-analysis included 11 full-text articles published from 2019 to 2022.

The main characteristics within the included studies are illustrated in Table 1. A total of 103 rodents (rats and mice) were included in the meta-analysis of MSC-EVs for periodontitis. With regard to the specific animals employed, six studies used Sprague–Dawley rats [8, 12, 13, 34, 38, 41], and five studies used mice (four studies with C57BL/6 [25, 39, 40, 42] and one study with CD-1 mice [43]). In terms of the periodontitis models, four studies utilized alveolar bone defect models that were created in the alveolar bone of first or second molars, with the outcomes related to changes in BV/TV [8, 12, 13, 34]; seven studies used the silk thread ligation method with or without coated bacteria, with the results related to the change in CEJ-ABC [25, 38–43]. With respect to the stem cell type (for EV isolation), three studies used human bone marrow mesenchymal stem cells (hMSCs) [12, 40, 41], two studies used stem cells from human exfoliated deciduous teeth (SHEDs) [34, 43], two studies used periodontal ligament stem cells (PDLSCs) [8, 13], two studies used dental pulp stem cells (DPSCs) [25, 42], one study used dental follicle cells (DFCs) [38], and one study used human gingiva tissue-derived MSCs (hGMSCs) [39].

**Table 1:** Characteristics of the included studies

Reference	Animal model	Evaluation index	EV source	EV isolation	EV size (nm)	Surface marker	Bracketry	Administration details	Dose	Signaling pathway
Chew 2019 [12]	SD rat	BV/TV	hMSC	Tangential flow filtration	100–200	CD81, ALIX, TSG101	Collagen sponge	Implanted into the bone defects	40 ug	AKT/ERK
Wu 2019 [34]	SD rat	BV/TV	SHED	Ultracentrifuge	50–200	CD9, CD81, TSG101	β-TCP	Implanted into the bone defects	100ug	AMPK
Zhao 2022 [13]	SD rat	BV/TV	PDLSC	Ultracentrifuge	90–150	CD9, TSG101	Hydrogel	Administrated into the alveolar bone defects		
Lei 2022 [8]	SD rat	BV/TV	PDLSC	1.Exo-quick Kit reagent 2.Ultracentrifuge	50–100	CD63, CD91, CD81	1. Matrigel 2. β-TCP	Injected into the bone defect	150 µg/µL	Wnt
Wei 2020 [43]	CD-1 mouse	CEJ-ABC	SHED	Ultracentrifuge	100	CD63	No	Injected into the buccal and lingual sides of the frst molar	20 µg/time, twice	Smad5/Runx2
Shimizu 2022 [25]	C57BL mouse	CEJ-ABC	HHH-DPC	Ultracentrifuge	50–200	CD9, CD63 CD81	No	Applied onto the silk ligature	7.5*10^8^particles/time, 3 times	
Liu 2021 [41]	SD rat	CEJ-ABC	hBMSC	Exosome Isolation™ reagent	50–200	CD63, TSG101	Hydrogel	Injected into periodontal pocket	500 µg/µL, once a week	OPG-RANKL-RANK
Shen 2020 [42]	C57BL mouse	CEJ-ABC	PDLSC	Ultracentrifuge	50–200	CD9, HSP70, CD63 CD81, TSG101	Hydrogel	Injected locally	50 µg	NF-κB p65 and p38 MAPK
Shi 2020 [38]	SD rat	CEJ-ABC	DFC	Exosome isolation reagent	30–150	CD63, TSG101	Hydrogel	Injected in periodontal pocket	50 µg/time, once a week	OPG/RANK/RANKL
Zhang 2021 [40]	C57BL mouse	CEJ-ABC	hMSC	Ultracentrifuge	50–200	CD63, CD9, TSG101	No	Injected into the palatal gingiva	50 µg/time, twice	miR-1246/Nfat5
Nakao 2021 [39]	C57BL mouse	CEJ-ABC	hGMSC	1.Exosome Isolation Kit 2. Ultracentrifuge	109 ± 3.1	CD9, CD63, CD81,	No	Injected into the palatal gingiva	20 µg	Wnt5a/RANKL

human mesenchymal stem cells, hMSC; stem cells from human exfoliated deciduous teeth, SHED; periodontal ligament stem cell, PDLSC

dental pulp stem cell, DPSC; human leukocyte antigen haplotype homo dental pulp cell, HHH-DP; dental follicle cell, DFC

human gingival tissue-derived mesenchymal stem cell, hGMSC

### Isolation and characterization of MSC-EVs

For the extraction method and characteristics of MSC-EVs, six studies utilized ultracentrifugation [13, 25, 34, 40, 42, 43], one study used exosome isolation reagent [38, 41], one study used Tangential flow filtration [12], and two studies used both ultracentrifugation and exosome isolation reagent [8, 39]. The size and quantity of EVs were assessed with the help of nanoparticle tracking analysis (NTA), dynamic light scattering analysis and NanoSight Analyzer, and the obtained EV diameter mostly ranged from 30 to 200 nm. In addition, the morphology of MSC-EVs was observed using transmission electron microscopy. Besides, EV surface markers in terms of CD9, CD63, CD81, ALIX, and TSG-101 were examined using western blotting or flow cytometry.

### Administration of MSC-EVs

For the administration methods, four studies utilized hydrogel as the EV carrier [13, 38, 41, 42], two studies used β-TCP [8, 24], one study used collagen sponge for EV delivery [12], and four studies did not use any bioactive materials [25, 39, 40, 43]. MSC-EVs with or without bioactive agents were placed into the alveolar bone defect or injected into the periodontal pocket directly. In addition, it was noteworthy that there were large differences in the total doses of MSC-EVs, varying from 20 to 100 µg or much more.

### Risk of bias

**Fig. 2:**
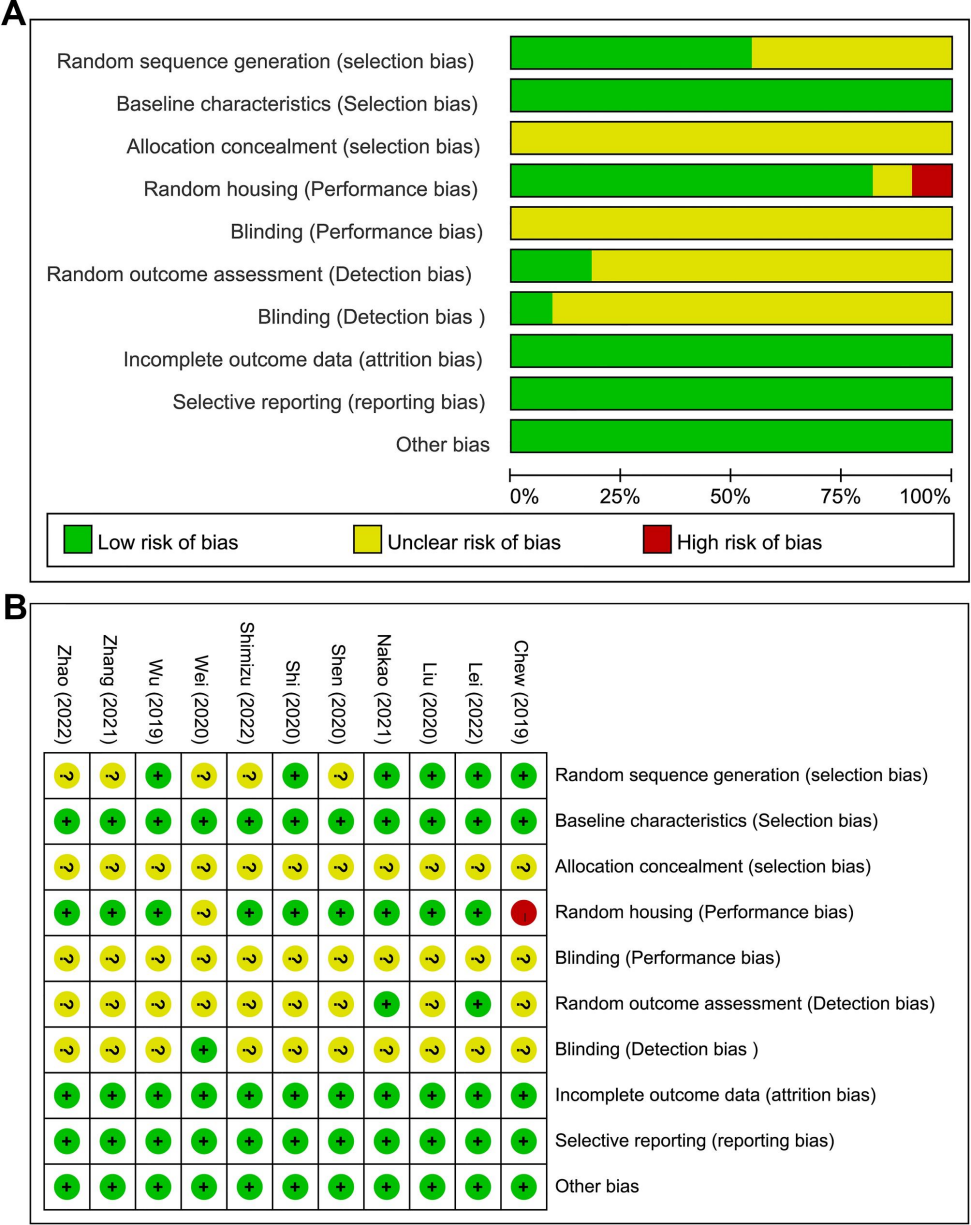
Risk of bias assessment. **A** Graph of the risk of bias for the included studies, **B** Graph of the risk of bias summary for the included studies

Quality assessments for all enrolled articles were performed. The details of the risk of bias are illustrated in Fig. 2. In specific, no study fulfilled all the criteria, while all studies exhibited similar baseline characteristics for the experimental and control groups. In particular, almost all of these studies were categorized as “unclear” for quality assessment of “allocation concealment” and “blinding for performance and detection” due to the absence of detailed information. Only one study was rated as “high” risk for their not random housing. In addition, no additional sources of bias were identified.

### Function of MSC-EVs on treatment of bone loss within periodontitis

**Fig. 3:**
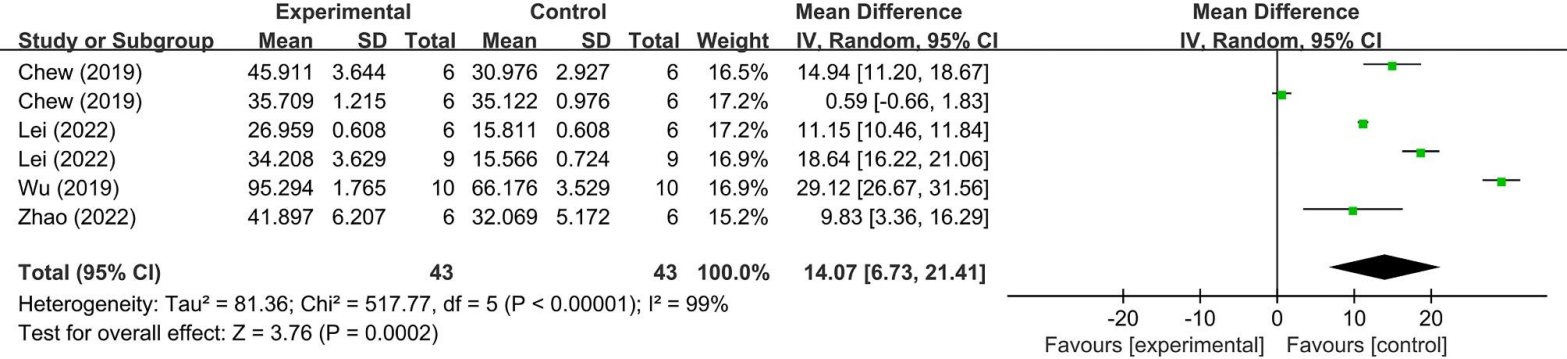
Forest plot demonstrated the mean effect size and 95% confidence interval (CI) for bone volume/total volume (BV/TV)

**Fig. 4:**
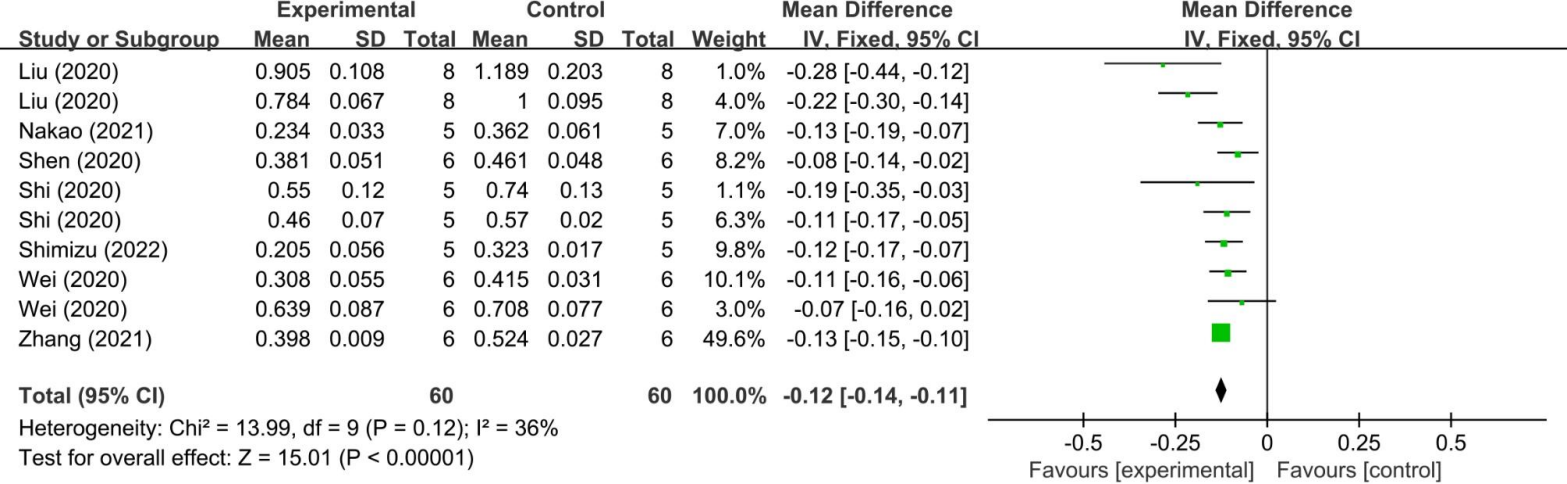
Forest plot shows the mean effect size and 95% confidence interval (CI) for distance between the cementoenamel junction and the alveolar bone crest (CEJ-ABC)

Based on the results of the present meta-analysis, MSC-EV treatment demonstrated a favorable effect to attenuate bone loss due to periodontitis, with a significant increase in WMD of BV/TV (14.07%; 95% CI = 6.73, 21.41%, *p* < 0.001, Fig. 3) and significant decrease of CEJ-ABC (-0.12 mm; 95% CI = -0.14, -0.11 mm, *p* < 0.001, Fig. 4) compared with the control groups, and a lower heterogeneity for CEJ-ABC outcomes (I^2^ = 36%). However, there was significant heterogeneity for BV/TV outcomes (I^2^ = 99%), which limits the strength of the conclusions regarding the function of MSC-EVs on treating bone defects within periodontitis.

**Fig. 5:**
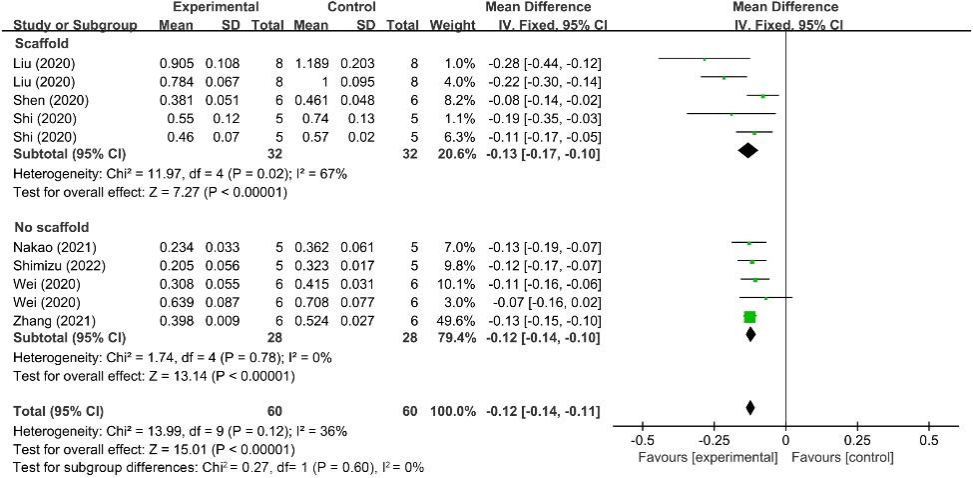
Subgroup analysis assessing the change in the distance between the cementoenamel junction and the alveolar bone crest (CEJ-ABC) in MSC-EV administration with or without bioactive scaffolds. MSC-EV, mesenchymal stem cell-derived extracellular vesicle

In addition, subgroup analysis for CEJ-ABC outcomes based on the EV delivery methods (in terms of with or without collagen sponge, β-tricalcium phosphate (β-TCP), hydrogel) was performed, and according to the results (Fig. 5), the decrease in WMD of CEJ-ABC between studies that with or without bioactive agents was not significant (*p* = 0.60).

### Signaling pathways impacted by MSC-EVs

**Fig. 6:**
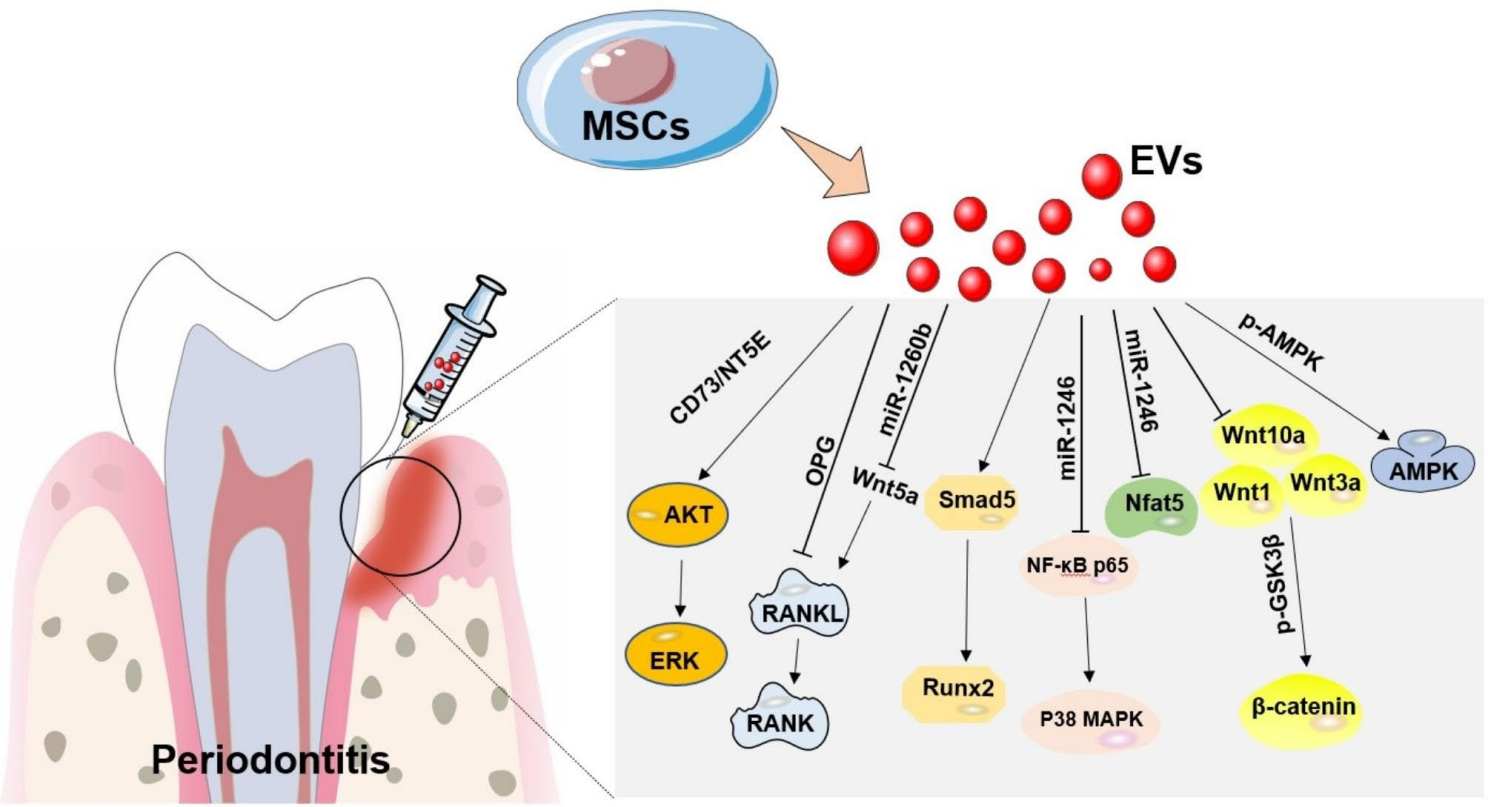
Mechanisms of MSC-EV treatment on periodontitis in included studies. Studies reported that signaling pathways including RANKL-RANK, Wnt, AMPK, AKT and ERK, NF-κB p65 and p38 MAPK, Smad5/Runx2, and the miR-1246/Nfat5 axis, were involved in the bone regeneration process mediated by MSC-EVs. MSC-EV, mesenchymal stem cell-derived extracellular vesicle. MSC-EV, mesenchymal stem cell-derived extracellular vesicle

The included studies reported that several pathways, including RANKL-RANK [38, 39, 41], Wnt [8], AMPK [34], AKT and ERK [12], NF-κB p65 and p38 MAPK [42], Smad5/Runx2 [43], and the miR-1246/Nfat5 axis [40] (Table 1; Fig. 6), may be involved in the alveolar bone regeneration process mediated by MSC-EVs.

### Sensitivity analysis

**Fig. 7:**
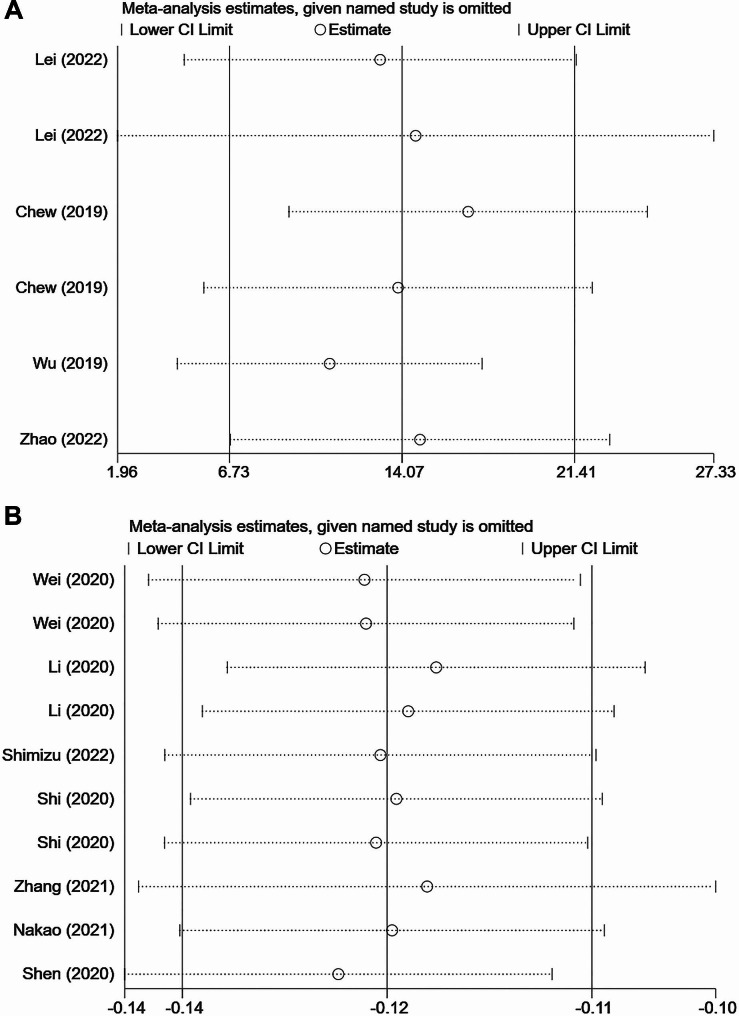
Sensitivity analysis of the studies included in bone volume/total volume (BV/TV) (**A**) and distance between the cementoenamel junction and the alveolar bone crest (CEJ-ABC) (**B**)

Sensitivity analysis was also performed to assess the stability of the present meta-analysis results. Figure 7 showed that the circles corresponding to the 11 included studies were located near the middle vertical line where the combined effect size located. It appeared that there were no studies had a significant impact on the combined effect size. Therefore, for the pooled MD, neither BV/TV outcome nor CEJ-ABC outcome were significantly affected by any study.

### Publication bias

**Fig. 8:**
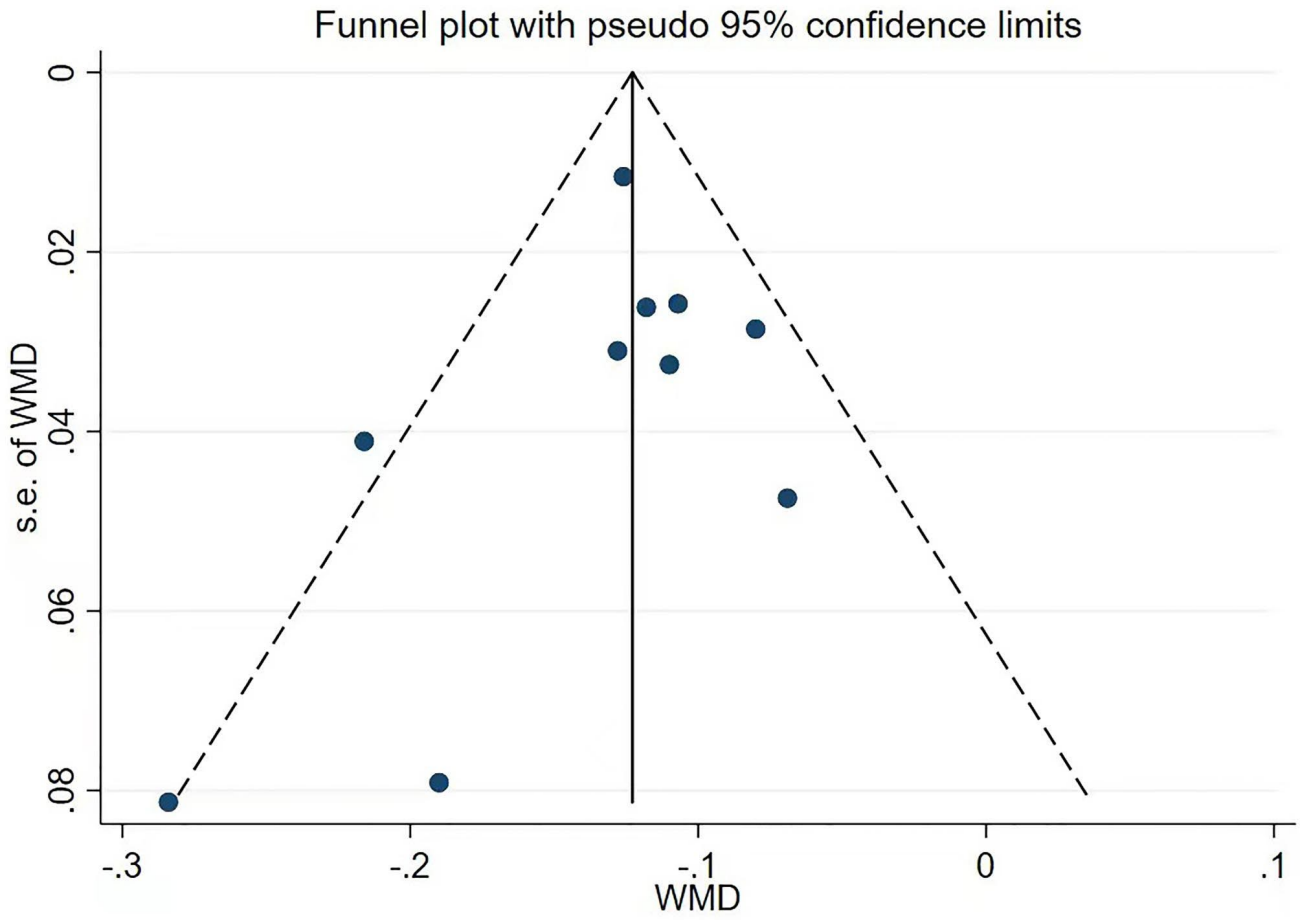
Publication-bias analysis results. Funnel plots for distance between the cementoenamel junction and the alveolar bone crest (CEJ-ABC)

Funnel plots and Egger’s tests were carried out to assess publication bias. As shown in Fig. 8; Table 2, the approximately symmetrical distributions of funnel plots and Egger’s test indicated that there was no significant publication bias for CEJ-ABC (*p* = 0.43). Funnel plot assessment was not carried out for BV/TV due to the limited number of studies (*n* = 4), while Egger’s test suggested an absence of publication bias for BV/TV (*p* = 0.55).

**Table 2:** Egger’s test of bone volume and total volume (BV/TV) and the distance between the cementoenamel junction and the alveolar bone crest (CEJ-ABC)

	*Egger’s test*			
	*Standard error*	*p*	*95% CI*	
BV/TV	7.363591	0.551	-15.65774	25.23148
ABJ	0.7802375	0.430	-2.448285	1.150177

## Discussion

There has been no effective strategy to cure inflammatory bone loss within periodontitis thus far. As key paracrine elements of MSCs, EVs were verified to possess the features of parent cells, and were considered to be an alternative for stem cell therapy due to their therapeutic potential in tissue regeneration [24, 25]. Under this context, increasing numbers of studies have been performed to explore the function of MSC-EVs in periodontitis animal models. The present meta-analysis of 11 studies afforded a relatively comprehensive summary of the efficacy of MSC-EVs in pre-clinical rodent model of periodontitis. Pooled analyses demonstrated that MSC-EVs could significantly promote alveolar bone regeneration within the inflammatory bone loss area, indicating that therapies based on MSC-EVs may yield new avenues for periodontitis in the clinic. However, considering the limited number of studies, more studies are needed to carry out to identify the beneficial effects of MSC-EVs in experimental periodontitis.

In our meta-analysis, BV/TV and CEJ-ABC outcomes were collected and analyzed to evaluate the efficacy of MSC-EVs in periodontitis. BV/TV and CEJ-ABC are the most commonly used indicators to evaluate the resorption and reconstruction performance of alveolar bone, especially for periodontitis animal models. BV/TV outcomes were demonstrated to accurately reflect cancellous and trabecular bone connectivity and bone volume, which are key indicators of bone quality [26]. While CEJ-ABC was defined as the distance between the cementoenamel junction and alveolar bone crest, which was also measured by micro-computed tomography, it truly reflects newly-formed bone in the inflammatory bone loss area due to periodontitis, thus carrying more clinical significance and reference value. Based on the meta-analysis results, much more new bone formed (BV/TV results) and the bone crest level was markedly elevated (CEJ-ABC results) in the MSC-EV groups compared to the control groups.

EV administrations with bioactive materials in terms of collagen sponge, β-TCP, and hydrogel were also investigated by several studies, and based on the enrolled studies, administration of EVs along with these bioactive materials exhibited significant pro-osteogenic potential in several studies although, pooled estimates of benefit from studies that MSC-EVs with bioactive agents were similar compared to studies of MSC-EVs without. Considering that the enhancement of EV retention has always been a challenging issue within the inflammatory bone defect environment, researchers have introduced bioactive materials into the EV research field. These materials were verified to provide mechanical support to the bone defect site, and facilitate the delivery and retention of EVs [27, 28]. In addition, bioactive scaffolds could permit the controlled release of EVs and further lead to more robust bone formation performance [29–31]. Matrigel is one of the most commonly used carriers for EVs, because its specific porose structure facilitates the prevision and delivery of EVs. In addition, it was reported that the controlled release of EVs can be achieved by regulating Matrigel degradation [32, 33]. A variety of studies have utilized TCP as the carrier for EVs to promote bone regeneration and repair, especially for bone defects resulted by inflammatory periodontitis. Based on these studies, EV-loaded TCP delivered a significantly beneficial function on alveolar bone regeneration within periodontitis [34, 35]. However, at present, controlling the loading capacity and the release speed of EVs remains intractable and requires further exploration [8]. Additionally, collagen sponges are considered to be a beneficial scaffold for EV administration in periodontitis and are widely utilized to maintain space along with other biologics for periodontal tissue regeneration [36, 37]. Although an obvious benefit from the use of biomaterial scaffolds was not identified in the present meta-analysis, their enhancing biological characteristics suggest that future study of these bioactive agents as EV carriers may be beneficial and worthwhile.

Within the studies identified in the present meta-analysis, some researchers have pre-treated MSCs before EV isolation, for instance, treating MSCs with LPS [38] or TNF-α [39], or culturing MSCs in a 3D system [40] before EV administration. Based on the reported outcomes, all of these preconditioning techniques resulted in significantly elevated bone crest levels, while due to the limited study number, a pooled sub-analysis was not performed. Additionally, investigators reported that MSC-EV administration in periodontitis led to changes in putative signaling pathways involved in osteogenesis. Three studies reported that MSC-EVs promote bone regeneration by RANKL-RANK signaling [38, 39, 41] and other pathways including Wnt signalling [8], AMPK signaling [34], AKT and ERK signaling [12], NF-κB p65 and p38 MAPK signaling [42], Smad5/Runx2 signaling [43], and the miR-1246/Nfat5 axis [40], were also reported to be involved in the bone regeneration process mediated by MSC-EVs. These pathways were previously demonstrated to be implicated in the bone injury healing process [26, 44]. Based on this context, modifying MSC-EVs with specific biologic signaling molecules (proteins, mRNA, miRNAs, lipids), and then deliver these biomolecules to target cells via modulating these above pathways is a topic worthy of further study.

Risk of bias of the enrolled studies was evaluated using the SYRCLE tool [22]. almost all of the studies were rated to be at an unclear risk in domains of selection (allocation concealment), performance (blinding), and detection (random outcome assessment, blinding), and one study was found to be at a high risk in performance (not random housing). Other significant bias including selection (baseline characteristics), attrition (incomplete outcome data), reporting (selective reporting) was not appreciated in our present studies. Future research should pay more attention to study designs to reduce the aforementioned potential risk of bias, which will increase our confidence in study results and contribute to performing a meaningful meta-analysis.

It is well known that meta-analyses of animal studies could offer guidance for basic research and clinical endeavors. To the best of our knowledge, this is the first meta-analysis of MSC-EVs in pre-clinical periodontitis experiments, which provide, to a certain degree, a summary of the efficacy of MSC-EV administration in periodontitis. However, several potential limitations should be considered. First, a relatively small number of studies within this meta-analysis, and the inevitable or accompanying presence of uncontrollable biases or uncertainties, may introduce potential influence and other bias, the explorations of the heterogeneity were not feasible for the BV/TV outcomes for the small number (*n* = 4) of included studies. Second, the 11 included studies were all rodent animal models; thus, more large-animal studies, such as pigs, should be considered in future research. Third, several factors in terms of EV source (stem cell types), EV isolation and storage methods (including freezing), EV administration dosages, timing and frequency may affect the efficacy of EVs in periodontitis, considering these above factors, the results of this study should be interpreted with caution.

For the future research, well designed large-scale animal studies should be performed, and large-animal periodontitis models should be established to confirm the function of MSC-EVs in treating periodontitis. Besides, the safety issues of MSC-EVs, especially the adverse events, should be recorded. To promote the translation of MSC-EVs into clinical practice, much progress has been made for the improvement of targeting functions of EVs by unique engineered strategies, for instance, loading or modifying EVs with RNAs, peptides or proteins via utilization of genetically engineered donor MSCs [45, 46]. For EVs bear enormous application potential as biological drug delivery vehicles, and with the development of tissue engineering, the clinical application of EVs will eventually be realized.

## Conclusion

Within the limitations of the present meta-analysis, MSC-EV administration delivered an effective function in the pre-clinical periodontitis animal models compared with the controls. While, the efficacy of MSC-EVs in periodontitis still needs to be further verified by large-scale animal studies as well as large-animal studies pre-clinically, with the aim of providing basis and clues for future clinical trials. Although, based on the present studies, the beneficial effect of scaffolds in treating bone loss was not confirmed, their specific biological properties suggest that these bioactive agents as EV vehicles may be of avail to treat periodontitis in future research.

### Electronic supplementary material

Below is the link to the electronic supplementary material.

Supplementary Material 1

## Data Availability

All data generated or analyzed during this study are included in this published article.

## Abbreviations

MSC: mesenchymal stem cell
EV: extracellular vesicle
CI: confidence interval
WMD: weighted mean difference
hMSC: human mesenchymal stem cell
SHED: stem cells from human exfoliated deciduous teeth
PDLSC: periodontal ligament stem cell
DPSC: dental pulp stem cell
HHH-DP: human leukocyte antigen haplotype homo dental pulp cell
DFC: dental follicle cell
hGMSC: human gingival tissue-derived mesenchymal stem cell
