# Supplementary material for: Mesenchymal stem cell-derived extracellular vesicles for treatment of bone loss within periodontitis in pre-clinical animal models: a meta-analysis
Source: BMC Oral Health. 2023 Sep 29;23:701. doi: 10.1186/s12903-023-03398-w (PMC10540343; doi:10.1186/s12903-023-03398-w)
Supplement: Supplementary file 1 — Supplementary Material 1 [file 12903_2023_3398_MOESM1_ESM.docx]

Table S1: the reasons for excluding the paper after title/abstract reading

|  | Inclusion criteria | Exclusion criteria |
| --- | --- | --- |
| Population | ·Animal models of periodontitis, with no restrictions on the animal species nor modeling methods | ·Studies that use animals with co-morbidiites, such as diabetes.  ·In vitro and in vivo studies in humans.  ·Only with in vitro results. |
| Intervention | ·Extracellular Vesicles derived from MSCs.  ·Modified or enriched MSC-EVs (enriched with miRNA, lipids, proteins, etc....). | ·EVs derived from other cells instead of MSCs or other tissues in periodontitis.  ·MSCs or their condition medium in periodontitis.  ·Any other intervention.  ·No control groups |
| Comparison | ·Studies with any comparator (sham-treated animals, animals with no treatment, placebo, etc.) will be considered. | ·Studies without a separate control group. |
| Outcome | ·Outcomes in terms of BV/TV, CEJ-ABC, and signaling pathways involved. | ·Not including the analysis outcome measures |
| Study design | ·Randomized, non-randomized or quasi-randomized in vivo studies | ·Non-comparative studies;  ·Studies reported only in the following forms: retrospective studies, and literature reviews; publications using replicated information; commentaries, |
